# Detection of patent foramen ovale in patients with ischemic stroke on prospective ECG-gated cardiac CT compared to transthoracic echocardiography

**DOI:** 10.1007/s00415-023-11688-0

**Published:** 2023-04-07

**Authors:** L. A. Rinkel, B. J. Bouma, S. M. Boekholdt, C. F. P. Beemsterboer, N. H. J. Lobé, L. F. M. Beenen, H. A. Marquering, C. B. L. M. Majoie, Y. B. W. E. M. Roos, A. van Randen, R. N. Planken, J. M. Coutinho

**Affiliations:** 1 Department of Neurology, Amsterdam University Medical Centres, University of Amsterdam, Location AMC, 1105 AZ Amsterdam, The Netherlands; 2 Department of Radiology and Nuclear Medicine, Amsterdam University Medical Centres, University of Amsterdam, Amsterdam, The Netherlands; 3 Department of Cardiology, Amsterdam University Medical Centres, University of Amsterdam, Amsterdam, The Netherlands; 4 Department of Biomedical Engineering and Physics, Amsterdam University Medical Centres, University of Amsterdam, Amsterdam, The Netherlands

**Keywords:** Stroke, Patent foramen ovale, Cardioembolism, CT, Echocardiography

## Abstract

**Background:**

Cardiac CT acquired during the acute stroke imaging protocol is an emerging alternative to transthoracic echocardiography (TTE) to screen for sources of cardioembolism. Currently, its diagnostic accuracy to detect patent foramen ovale (PFO) is unclear.

**Methods:**

This was a substudy of Mind the Heart, a prospective cohort in which consecutive adult patients with acute ischemic stroke underwent prospective ECG-gated cardiac CT during the initial stroke imaging protocol. Patients also underwent TTE. We included patients < 60 years who underwent TTE with agitated saline contrast (cTTE) and assessed sensitivity, specificity, negative and positive predictive value of cardiac CT for the detection of PFO using cTTE as the reference standard.

**Results:**

Of 452 patients in Mind the Heart, 92 were younger than 60 years. Of these, 59 (64%) patients underwent both cardiac CT and cTTE and were included. Median age was 54 (IQR 49–57) years and 41/59 (70%) were male. Cardiac CT detected a PFO in 5/59 (8%) patients, 3 of which were confirmed on cTTE. cTTE detected a PFO in 12/59 (20%) patients. Sensitivity and specificity of cardiac CT were 25% (95% CI 5–57%) and 96% (95% CI 85–99%), respectively. Positive and negative predictive values were 59% (95% CI 14–95) and 84% (95% CI 71–92).

**Conclusion:**

Prospective ECG-gated cardiac CT acquired during the acute stroke imaging protocol does not appear to be a suitable screening method for PFO due to its low sensitivity. Our data suggest that if cardiac CT is used as a first-line screening method for cardioembolism, additional echocardiography remains indicated in young patients with cryptogenic stroke, in whom PFO detection would have therapeutic consequences. These results need to be confirmed in larger cohorts.

**Supplementary Information:**

The online version contains supplementary material available at 10.1007/s00415-023-11688-0.

## Introduction

Patent foramen ovale (PFO) is present in approximately a quarter of the general population, but is considerably more common in patients with cryptogenic stroke [1–3]. PFO closure is indicated in patients younger than 60 years with cryptogenic stroke to reduce stroke recurrence. Although transoesophageal echocardiography (TEE) has been considered the gold standard for diagnosis of PFO, up to one third of stroke patients cannot undergo TEE due to the severity of their stroke, dysphagia, excessive gag reflexes or refusal. Moreover, use of sedation makes the performance of the Valsalva maneuver during TEE more difficult [4, 5]. Previous studies have suggested that transthoracic echocardiography with agitated saline contrast (cTTE) has a similar detection rate for PFO compared to TEE when performed by skilled operators [6, 7]. Therefore, cTTE is most commonly used to screen for PFO [8, 9].

Cardiac CT acquired during the acute stroke imaging protocol is an emerging alternative to TTE to screen for cardioembolism, and a recent study showed that cardiac CT has a higher diagnostic yield than TTE for the detection of high-risk sources of embolism [10]. Currently, the diagnostic accuracy of cardiac CT acquired during the acute stroke imaging protocol to detect PFO is unclear. We, therefore, aimed to assess the sensitivity, specificity, positive predictive value, and negative predictive value of prospective ECG-gated cardiac CT in acute ischemic stroke patients to determine whether it is a suitable screening method for the detection of PFO.

## Methods

This was a substudy of the Mind the Heart study, a prospective single-center cohort study in which the diagnostic yield of cardiac CT acquired during the acute stroke work-up was evaluated. Between 2018 and 2020, we included consecutive adult patients with acute ischemic stroke who were potentially eligible for reperfusion therapy (intravenous thrombolysis [IVT] or endovascular treatment [EVT]) at the time of admission (i.e., patients with acute onset neurological symptoms that developed less than 24 h ago). Patients who underwent prospective ECG-gated cardiac CT were triggered to scan during end diastole as part of the initial stroke imaging protocol. Cardiac CT was acquired immediately following non-contrast-enhanced CT of the brain, CT perfusion, and non-gated CT-angiography of the aortic arch, cervical and intracranial arteries. Patients also underwent routine stroke work-up, including TTE. TEE was not routinely performed. Additional details of the study have been published [10]. For the current study, we included all patients who underwent TTE with agitated saline contrast, which was indicated in patients < 60 years in the Mind the Heart study.

Details of the imaging protocol have been published [11]. Presence of PFO was assessed on CT and cTTE according to predefined criteria [11]. On CT, a PFO was defined as a crypt-shaped contrast jet from the left atrium to the right atrium toward the vena cava, or an atrium septum discontinuity [12, 13]. All CT images were systematically assessed by a cardiac radiologist. On cTTE, a PFO was defined as the appearance of microbubbles in the left atrium within 3–6 cardiac beats after opacification of the right atrium [8]. All images were assessed by a cardiologist. During assessment, the cardiac radiologists and cardiologists were blinded to each other’s results. In case of indeterminate results on cardiac CT or cTTE (i.e., due to insufficient image quality), PFO was scored as not being present.

### Statistical analysis

Using TTE with agitated saline injection as reference standard, we assessed the sensitivity, specificity, positive predictive value, and negative predictive value for detection of PFO on cardiac CT. Analyses were performed using R software, version 4.0.3 (R foundation for Statistical Computing 2018).

### Sensitivity analyses

We determined the sensitivity of cardiac CT when limiting PFO’s to the criteria of the CLOSE study, since these PFO’s are potentially relevant to treat. The criteria of the CLOSE study were: no identifiable cause other than a PFO with an associated atrial septal aneurysm or large interatrial shunt (appearance of more than 30 microbubbles in the left atrium within 3 cardiac cycles after opacification of the right atrium). [14]

We assessed whether the results were consistent when also including patients who were 60 years or older and underwent cTTE.

## Results

**Fig. 1:**
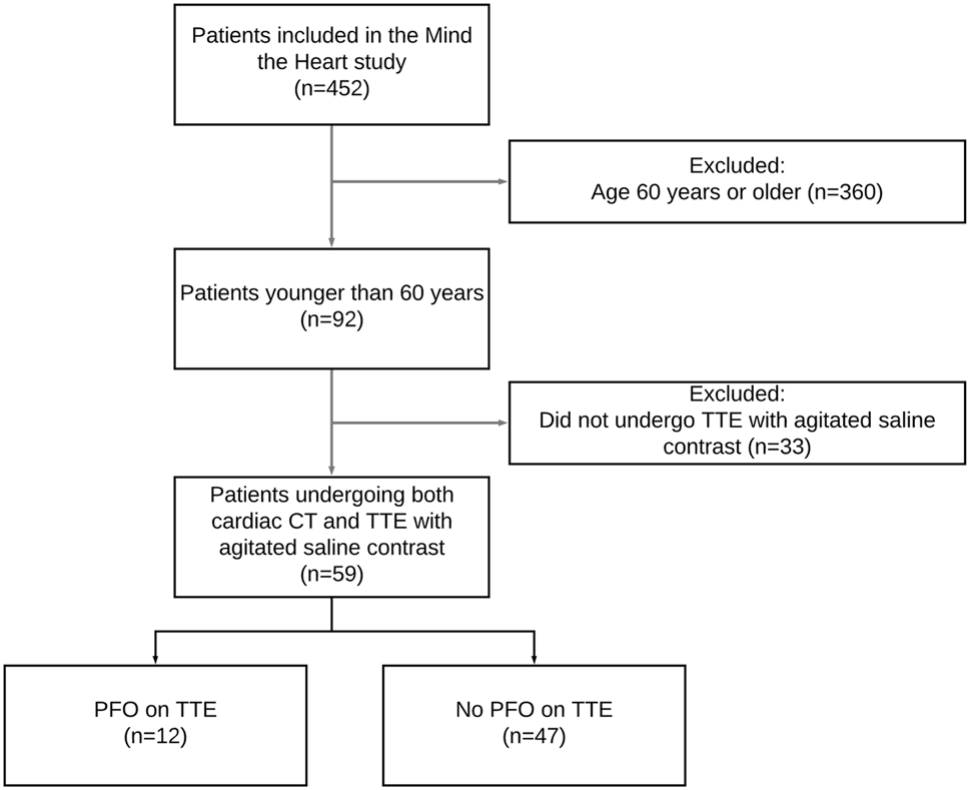
Flowchart of patients. *TTE* transthoracic echocardiography, *PFO* patent foramen ovale. The reasons for 33 patients not undergoing cTTE were: no echocardiography performed (12 patients, 4 because they died before TTE could be acquired and 8 because TTE in the outpatient setting was too burdensome), echocardiography without contrast performed in another hospital (6 patients), clear other cause of stroke and therefore detection of PFO deemed inconsequential by treating physician (6 patients), failure to acquire IV access (1 patient), and unclear reasons (8 patients)

Of 452 patients included in the Mind the Heart study, 92 (20%) patients were younger than 60 years. Of these, 59 (64%) underwent cTTE and were included in the current study (Fig. 1).

Median age was 54 (interquartile range [IQR] 49–57) years and 41 (70%) were male (Table 1). In total, 33/59 (56%) patients had cryptogenic stroke and their median Risk of Paradoxical Embolism score was 6 (IQR 6–7). Stroke etiology in the remaining patients was large-artery atherosclerosis in five, cardioembolism in eight, small vessel disease in seven, and other determined in six. There were four indeterminate results on CT, all due to low scan quality, and none on cTTE. Median time difference between cardiac CT and cTTE was 2 (IQR 0–16) days.

**Table 1:** Baseline characteristics

	Study population, *N* = 59
Median age (IQR)—year	54 (49–57)
Male sex—no. (%)	41 (70)
Median systolic blood pressure (IQR)—mmHg^a^	146 (131–172)
Median NIHSS score (IQR)	4 (3–13)
Medical history—no. (%)	
Previous ischemic stroke	4 (7)
Transient ischemic attack	2 (3)
Atrial fibrillation	2 (3)
Diabetes mellitus	5 (9)
Hypertension	12 (20)
Hypercholesterolemia	2 (3)
Smoking^a^	29 (51)
Malignancy	3 (5)
Myocardial infarction	3 (5)
Median pre-stroke modified Rankin scale score (IQR)	0 (0–0)
Medication use—no. (%)	
Anticoagulation	1 (2)
Antiplatelet	9 (15)
Anti-hypertensive drugs	14 (24)
Statin	8 (14)
Intracranial large vessel occlusion—no. (%)	24 (41)
Reperfusion therapy—no. (%)	
IV thrombolysis	25 (42)
Endovascular thrombectomy	20 (34)
Process time, median duration (IQR)	
Onset-to-door time^b^—min	148 (60–517)
Door-to-needle time^c^—min	41 (28–50)
Door-to-groin time—min	62 (55–75)
Stroke etiology	
Large artery atherosclerosis	5 (8)
Cardioembolic	8 (14)
Small vessel disease	7 (12)
Other determined	6 (10)
Cryptogenic	33 (56)
RoPE score, median IQR*	6 (6–7)

*IQR* interquartile range

Missing values, *n* (%): ^a^ 2 (3), ^c^ 1 (5)

*Risk of Paradoxical Embolism score, only determined for patients with cryptogenic stroke

**Fig. 2:**
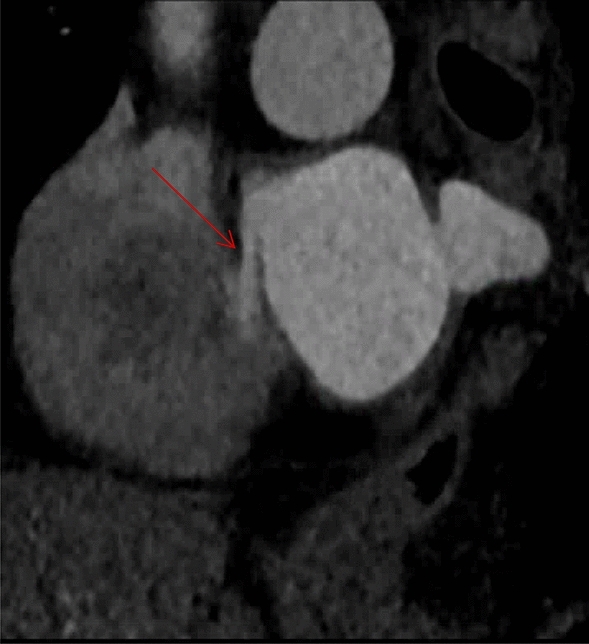
Oblique sagittal reformation showing a crypt-shaped contrast jet from the left atrium to the right atrium toward the vena cava (red arrow) on cardiac CT, indicative of a PFO which was later confirmed on TTE with agitated saline contrast

**Fig. 3:**
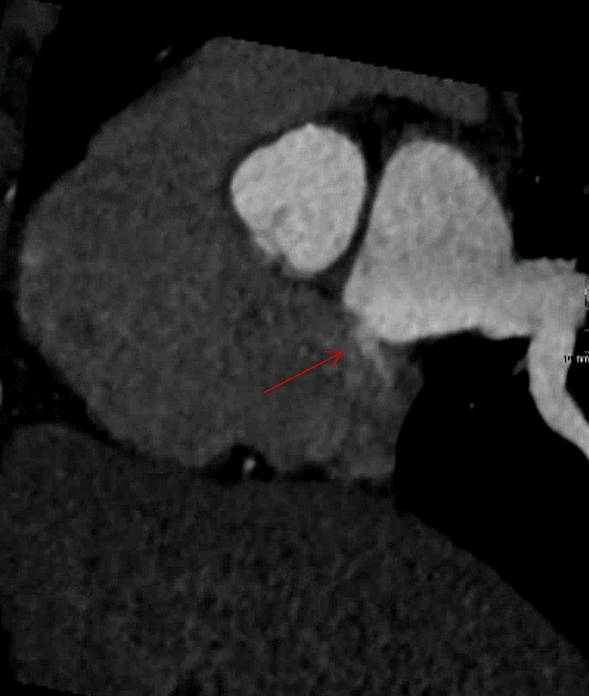
Cardiac CT showing a left-to-right contrast jet (red arrow) scored as PFO. TTE with agitated saline contrast did not detect a PFO in this patient

Cardiac CT detected a PFO in 5/59 (8%) patients, 3 of which were confirmed on cTTE (Table 2, Figs. 2, 3). cTTE detected a PFO in 12/59 (20%) patients. This resulted in a sensitivity of cardiac CT of 25% (95% confidence interval [95% CI] 5–57%) and specificity of 96% (95% CI 85–99%, Table 3). The positive and negative predictive values were 59% (95% CI 14–95) and 84% (95% CI 71–92), respectively.

**Table 2:** Diagnosis of PFO on prospective ECG-gated cardiac CT vs transthoracic echocardiography with agitated saline contrast

	PFO present on TTE	PFO absent on TTE	Total
PFO present on cardiac CT	3	2	5
PFO absent on cardiac CT	9	45	54
Total	12	47	59

**Table 3:** Sensitivity, specificity, positive predictive value, and negative predictive value of prospective ECG-gated cardiac CT vs transthoracic echocardiography with agitated saline contrast for detection of PFO

	Value (%) (95% confidence interval)
Sensitivity	25 (5–57)
Specificity	96 (85–99)
Positive predictive value	59 (14–95)
Negative predictive value	84 (71–92)

Both cardiac CT and cTTE did not detect an atrial septal aneurysm (ASA) in any of the patients with a PFO. One patient with a PFO had a jump-rope interatrial septum detected on echocardiography, though this did not fulfill the criteria for presence of an atrial septal aneurysm (protrusion of > 10 mm beyond the plane of the atrial septum).

Of the 12 patients with a PFO on cTTE, 3 had another identifiable cause of their stroke. Of the remaining nine patients, two did not have a large interatrial shunt according to the CLOSE criteria (< 30 bubbles), meaning that 7 PFO’s fulfilled the criteria of the CLOSE study. Cardiac CT detected a PFO in two of seven of these patients, indicating a similar sensitivity (28%) in this subgroup of patients with a PFO. Results were similar when also including the five patients of 60 years and older who underwent cTTE (supplemental material Tables 1, 2).

Three patients underwent TEE and a PFO was detected on TEE in two of them. Indications for TEE were: assessment if the PFO was eligible for device closure in one, confirmation of PFO detected on cTTE in one, and assessment of the left atrial appendage in the third. No patients underwent transcranial Doppler (TCD) for detection of right–left shunting.

## Discussion

In this study, prospective ECG-gated cardiac CT acquired during the acute stroke protocol had a low sensitivity for the detection of PFO in patients with acute ischemic stroke. These data suggest that cardiac CT fails to detect the majority of PFO’s.

The low sensitivity of prospective ECG-gated cardiac CT may be explained by several reasons. First, detection of PFO on CT and TTE is based on different principles. cTTE is used to identify a temporarily right-to-left shunt during the Valsalva maneuver which results in a brief moment of right atrial pressure exceeding the left atrial pressure. In contrast, CT is used to diagnose PFO detecting a left-to-right shunting during rest. The shunt flow through a PFO is determined by differences in pressure between the left and right atrium. Pressure differences between the left atrium and right atrium are the largest during end systole, isovolumetric relaxation, and early diastole [15], while cardiac CT was triggered to scan only during end diastole in our cohort. As such, the false negative findings on CT could be due to equivalent pressures in the left and right atrium at the moment of image acquisition. Moreover, left-to-right shunting may not occur in all patients with a PFO [16–18], indicating that these PFO’s may be undetectable on cardiac CT. Furthermore, the applied single-phase prospective ECG-gated cardiac CT acquisition does not allow for dynamic assessment of the heart since only a single phase of the cardiac cycle is scanned, which may further hamper detection of PFO’s. Previous studies compared retrospective ECG-gated cardiac CT, in which the heart is scanned throughout the entire cardiac cycle and images are reconstructed retrospectively, with TEE for the detection of PFO and these studies reported a higher sensitivity and specificity of cardiac CT [15, 19]. However, since more radiation is required for retrospective gated cardiac CT, we decided on prospective ECG gating for our acute stroke imaging protocol.

Although studies have shown that TEE and TCD have a higher diagnostic accuracy than TTE [20], TTE is often the first-line screening method for PFO [8]. Previous studies comparing TEE with TTE and TCD for the detection of PFO reported reasonable sensitivity and specificity for TTE, with the discrepancy between the modalities mainly being driven by false negatives on TTE with very few false positives [21]. Hence, it is likely that if we would have compared prospective gated cardiac CT to TEE or TCD, rather than TTE, the sensitivity of cardiac CT would only have been lower.

A previous meta-analysis of individual patient data of six randomized controlled trials showed that the PFO-Associated Stroke Causal Likelihood (PASCAL) classification performs best in identifying patients who may benefit most from PFO closure [22]. The PASCAL score combines the Risk of Paradoxical Embolism (RoPE) score with additional assessment of high-risk features, either large size of the shunt or presence of atrial septum aneurysm. Although the presence of atrial septum aneurysm can be assessed on prospective ECG-gated cardiac CT, it does not allow for reliable quantification of PFO size. Moreover, cardiac CT detected only 22–73% of atrial septum aneurysms which were diagnosed on echocardiography in previous studies [23, 24]. This provides another argument that if prospective ECG-gated cardiac CT is implemented as a screening method for cardioembolic sources, additional echocardiography is required in patients in whom detection of a PFO has therapeutic consequences.

We were unable to assess the sensitivity of cardiac CT acquired during the acute phase for detection of atrial septal defects (ASD), since none were diagnosed on cTTE. On echocardiography, ASD is diagnosed by detecting a left-to-right shunt. On CT, PFO is differentiated from ASD based on the direction of the left-to-right contrast agent jet, which is toward the vena cava inferior for PFO and perpendicular to the atrial septum for ASD. A previous study showed that retrospective ECG-gated cardiac CT detected all three ASD’s which were diagnosed on TEE [24]. More data are needed to determine whether cardiac CT during the stroke imaging protocol is a suitable screening technique for the detection of ASD.

Prospective gated cardiac CT has a higher diagnostic yield for various high-risk structural sources of embolism than TTE, such as cardiac thrombi [10]. However, our findings suggest that if prospective cardiac CT is implemented in the initial stroke imaging protocol to screen for cardiac structural sources of embolism, echocardiography or TCD will remain essential for PFO detection.

This study has several limitations. First, the sample size is limited, although it is one of the largest studies to date comparing prospective cardiac CT acquired in the acute phase of ischemic stroke to TTE with agitated saline for the detection of PFO. Second, we did not compare CT with TEE or TCD which have a higher diagnostic yield for PFO detection. Third, this was a single-center study, which limits the generalizability of the results. Finally, we cannot exclude that selection bias occurred since 33 patients in our cohort who were younger than 60 years did not undergo cTTE.

## Conclusion

Prospective ECG-gated cardiac CT acquired during the acute stroke imaging protocol does not appear to be a suitable screening method for PFO due to its low sensitivity. Our data suggest that if cardiac CT is used as a first-line screening method for cardioembolism, additional echocardiography remains indicated in young patients with cryptogenic stroke, in whom PFO detection would have therapeutic consequences. These results need to be confirmed in larger cohorts.

## Supplementary Information

Below is the link to the electronic supplementary material.

Supplementary file1 (DOCX 16 KB)

## Data Availability

Anonymized data not published within this article will be made available on reasonable request and requires approval by the privacy officer of Amsterdam UMC for GDPR compliance.
