# Supplementary material for: Detection of patent foramen ovale in patients with ischemic stroke on prospective ECG-gated cardiac CT compared to transthoracic echocardiography
Source: J Neurol. 2023 Apr 7;270(7):3537–42. doi: 10.1007/s00415-023-11688-0 (PMC10266986; doi:10.1007/s00415-023-11688-0)
Supplement: Supplementary file 1 — Supplementary file1 (DOCX 16 KB) [file 415_2023_11688_MOESM1_ESM.docx]

**Supplemental materials for:**

Detection of patent foramen ovale in patients with ischemic stroke on prospective ECG-gated cardiac CT compared to transthoracic echocardiography

L.A. Rinkel, MD,^a^ B.J. Bouma, MD, PhD,^b^ S.M. Boekholdt, MD, PhD,^b^ C.F.P. Beemsterboer, Bsc,^a^ N.H.J. Lobé, BSc,^b^ L.F.M. Beenen, MD, PhD,^b^ H.A. Marquering, PhD,^b,d^ C.B.L.M. Majoie, MD, PhD,^b^ Y.B.W.E.M. Roos, MD, PhD,^a^ A. van Randen, MD, PhD,^b^ R.N. Planken, MD, PhD,*^b^ and J.M. Coutinho, MD, PhD*^a^

* Shared last authorship

**Supplemental table 1:** Diagnosis of PFO on prospective ECG-gated cardiac CT vs transthoracic echocardiography with agitated saline contrast including patients of 60 years and older.

|  | PFO present on TTE | PFO absent on TTE | Total |
| --- | --- | --- | --- |
| PFO present on Cardiac CT | 4 | 2 | 6 |
| PFO absent on Cardiac CT | 9 | 49 | 58 |
| Total | 13 | 51 | 64 |

**Supplemental table 2:** Sensitivity, specificity, positive predictive value and negative predictive value of prospective ECG-gated cardiac CT vs transthoracic echocardiography with agitated saline contrast for detection of PFO including patients of 60 years and older.

|  | Value (%)  (95% confidence interval) |
| --- | --- |
| Sensitivity | 31  (9–61) |
| Specificity | 96  (87–99) |
| Positive Predictive Value | 66  (22 to 96) |
| Negative Predictive Value | 84  (73 –93) |
